# Protective Effects and Mechanisms of MicroRNA-182 on Oxidative Stress in RHiN

**DOI:** 10.1515/biol-2019-0045

**Published:** 2019-08-28

**Authors:** Lihua Li, Wenna Peng, Xiangrong Tian

**Affiliations:** 1 Colleges of Medicine, Jishou University, Jishou, Hunan Province, P.R. China; 2 Department of Rehabilitation Medicine, The Second Xiangya Hospital of Central South University, Changsha, Hunan Province, P.R. China; 3 Biology and Environmental Sciences, Jishou University, Jishou, Hunan Province, P.R. China

**Keywords:** miR-182, rat hippocampal neurons, oxidative stress

## Abstract

To explore protective effects and related mechanisms of microRNA-182 (miR-182) on oxidative stress in rat hippocampal neurons (RHiN), RHiN cells. As the results, the survival rate and superoxide dismutase levels in H_2_O_2_ group were significantly lower than H_2_O_2_+miR-182 group (all P<0.05). The malondialdehyde levels and apoptosis rate in H_2_O_2_+miR-182 group were significantly lower than H2O2 group (all P<0.05). The mRNA levels and expression levels of mTOR and PI3K in H_2_O_2_+miR-182 group were higher than those in H_2_O_2_ group (both P<0.05). The experiment of cerebral ischemic oxidative stress model rats showed that the survival rate, apoptosis rate, malondialdehyde and superoxide dismutase levels in miR-182 group were better than model control group. The positive staining intensity of phosphoinositide 3-kinase (mTOR) and phosphoinositide 3-kinase (PI3K) in model control group were significantly lower than miR-182 group (all P<0.05). Increased levels of miR-182 can reduce the damage of H2O2 treatments in RHiN cells. Oxidative stress is decreased in the neuronal cells possibly by activation of the PI3K-AKT-mTOR pathway.

## 1 Introduction

Oxidative stress mainly refers to the large number of reactive oxygen species (ROS), reactive nitrogen species (RNS) and other free radicals of the oxidation system. These species are generated due to pathological stimulation and promote lipid peroxidation of the cell membrane system, leading to decline in DNA, protein, and other macromolecular activities [1]. The nerve tissue in the hippocampus is involved in memory and neural information processing. This part of the brain tissue is injured by oxidative stress, which may lead to neurological diseases in patients [2]. Studies have discovered that brain tissue of patients with vascular dementia and cerebral infarction exhibits cerebral ischemia, hypoxia and other inflammatory symptoms. The levels of ROS, nitric oxide synthase (NOS) and other free radicals in the brain tissue are increased as a result. The result of these processes is an aggravation of the patientsʼ oxidative stress [3]. Gong et al. have found that free radicals in the brain tissue of patients with senile dementia are high, causing lipid peroxidation in hippocampal neurons leading to their dysfunction. Outcomes of such damage are manifest as a poorer quality of life and a decrease in mental processes [4].

microRNA-182 (miR-182) is a single-stranded noncoding RNA consisting of 23 nucleotides. It was initially discovered in murine retinal tissue, and was found to regulate retinal sensitization in mice [5]. Studies have shown that miR-182 has a wide range of applications in biological systems. Metabolic and cellular division functions are regulated by control of target gene expression levels, and are closely related to many diseases, including those associated with nerve injury, tumor development, and depression [6]. Kho et al. have found that miR-182 can reduce ROS levels of cardiomyocytes in rats as well as normalise the apoptosis rate of the cardiomyocytes [7]. At present, there are few studies which detail the antioxidative protective effects of miR-182 on hippocampal neurons. The aim of this study is to explore protective effects and related mechanisms of miR-182 on oxidative stress in rat hippocampal neurons (RHiN).

## 2 Materials and methods

### 2.1 Cell groupings and culture

RHiN cells were purchased from Shanghai YS Industrial Co., Ltd., China. Dulbeccoʼs Modified Eagle Medium (DMEM) culture media, consisting of 10% fetal bovine serum, penicillin (150 U/mL), and streptomycin (120 U/mL), were all purchased from Shanghai Beinuo Biological Technology Co., Ltd., China. Cell culture groups were defined by their treatments: RHiN in blank control group were routinely cultured with DMEM media culture. Hippocampal neurons in miR-182 group were transfected with miR-182 (Guangzhou RiboBio Co., Ltd., China) according to Lipofectamine 2000 Kit instructions (Beijing Solarbio Science & Technology Co., Ltd., China). A total of 200 μmol/L of H_2_O_2_ solution (Sangon Biotech (Shanghai) Co., Ltd., China) was added to the culture medium of the hippocampal cells in H_2_O_2_ group. A total 200 μmol/L of H_2_O_2_ solution and 80 μmol/L of mTOR protein (Beijing Biolab Technology Co., Ltd., China) were added to the culture medium of the hippocampal cells in mTOR+H_2_O_2_ group. A total 8 μmol/L of rapamycin solution (Sigma Co., Ltd., Germany) was added to the culture medium of the hippocampal cells in mTOR inhibitor group. A total 10 μmol/L of MHY1485 solution (MedChemExpress Co., Ltd., USA) was added to the culture medium of the hippocampal cells in mTOR activator group. Hippocampal neurons in H_2_O_2_+miR-182 group were transfected with miR-182 and supplemented with 200 μmol/L H_2_O_2_ solution. All cell cultures were placed in an incubator at 37°C, with 5% CO_2_.

### 2.2 Detection of activity and apoptosis levels in RHiN cells

Cultured cells at exponential growth phase were seeded into 96-well culture plates, 1 x10_4_ cells per well. Cells were cultured for 24 h according to treatment groups, washed 3 times with ice-cold phosphate buffer saline (PBS), followed by addition of 25 μL of MTT (5 g/L) (Beijing Biolab Technology Co., Ltd., China) to each well. Cells were cultured for an additional 4 h. Following the incubation with MTT, the supernatant was discarded and 150 μL dimethylsulfoxide (DMSO, Shanghai Yeasen Biotechnology Co., Ltd., China) was added to each well for dissolution. A micro oscillator was used for 10 min to dissolve completely any crystals formed during the incubation. The optical density (OD) at 492 nm was determined using microplate reader (Shanghai Flash Spectrum Biological Technology Co., Ltd., China). The method for calculating the cell survival rate is as follows: survival rate = (OD in the treatment group / OD in the blank control group) x 100%.

Flow cytometry was used to analyze the effect of siRNA transfection. RHiN treatment groups were cultured to logarithmic phase. These cells were digested for 5 min using 0.25% pancreatin (Shanghai Yeasen Biotechnology Co., Ltd., China), and then centrifuged for 10 min at 2,000 rpm/min. After collecting cells and washing twice with ice-cold PBS, 600 μL ethanol was added to cells, and placed at -4°C overnight. Cells were washed twice more with PBS and 350 μL binding buffer was added to resuspend cells. Annexin V-Enhanced Green Fluorescent Protein (EGFP) (10 μL, Shanghai Yubo Biological Technology Co., Ltd., China) was added for mixing. Propidium iodide (PI) (5 μL) staining solution was added, incubated for 10 min at room temperature, and flow cytometry (Beckman Coulter Co., Ltd., USA) was carried out to detect cellular apoptosis levels. The excitation wavelength was set to Ex=488 nm, and the emission wavelength was set to Em=530 nm.

### 2.3 Detection of superoxide dismutase (SOD) and malondialdehyde (MDA) levels in RHiN cells

Exponential phase cells were seeded into 96-well culture plates, 1x10_4_ cells per well. Cells were cultured for 24 h according to treatment groups. Following cell collection, radioimmunoprecipitation assay (RIPA) buffer (Shanghai Yeasen Biotechnology Co., Ltd., China) was added and cells were incubated for 10 min at 4°C, then centrifuged for 5 min at 12,000 rpm. Supernate was taken in low-temperature preservation. The SOD levels of RHiN were detected by enzyme-linked immuno sorbent assay (ELISA) according to the manufacturer’s instructions (Shanghai Jianglai Biotech Co., Ltd., China). The OD value of each well was determined at 450 nm. Thiobarbituric acid (TBA) was utilized to detect the levels of MDA, according to the MDA Assay Kit instructions (Shanghai Yaji Biotech Co., Ltd., China). TBA was purchased from Beijing Biolab Technology Co., Ltd., China. The OD value of the test sample supernatant was determined at 532 nm.

### 2.4 Reverse transcription-polymerase chain reaction (RT-PCR) detection of PI3K and mTOR mRNA levels

RHiN cells were cultured to logarithmic phase after appropriate treatments. Following cell collection, total RNA was extracted by TRIzol reagent (obtained from Shanghai Yeasen Biotechnology Co., Ltd., China). A nanodrop spectrophotometer (Thermo Fisher Scientific Inc., USA) was used for verifying the quality of RNA samples. Complementary DNA (cDNA) libraries were synthesized by RNA reverse transcription kit (Shanghai Yeasen Biotechnology Co., Ltd., China). Reaction conditions were set at 42°C for 15 min and 80°C for 5 min. The product was stored at 4°C. Glyceraldehyde-3-phosphate dehydrogenase (GAPDH) was used as the reference gene for the purpose of this study. Primer 5.0 (Suzhou Genewiz Biology Company, China) was used to design forward and reverse amplification primers. For PI3K, the upstream primer sequence was 5ʼ-CACTGGCATCCTCACTCAC-3ʼ and downstream primer sequence was 5ʼ-CTGACTGACTCACTGC-3ʼ. For mTOR, the upstream primer sequence was 5ʼ-CGTCAACCATCCATGTAC-3ʼ and the downstream primer was 5ʼ-CGTACTGCATGCACTCATGCC-3ʼ. For GAPDH, the upstream primer was 5ʼ-TCACTGCACTGCA-CTGCAC-3ʼ, and the downstream primer was 5ʼ-CGCTGACTGACGTCAC-3ʼ. Reaction conditions were initiated at 95°C for 30 s, 95°C for 5 s, 62°C for 20 s, 72°C for 30 s, and the reaction was run for 32 cycles. The 2^-ΔΔCt^ was used to calculate relative expression levels of target genes.

### 2.5 Western blot analysis of PI3K and mTOR protein expression

RHiN cells in the logarithmic phase were collected. RIPA lysis buffer was used to treat cells for 15 min prior to total protein extraction. Protein concentration was determined using the bicinchoninic acid assay (BCA assay). The protein sample solution and 5**×** protein loading buffer (obtained from Beyotime Biotechnology Company, China) were mixed. After boiling for 6 min, samples were cooled to room temperature. Denatured protein (15 μg) was added to sample wells of 10% of sodium dodecyl sulfate polyacrylamide gel electrophoresis (SDS-PAGE) gels, and proteins were electrophoresed. Following electrophoresis, total proteins were transferred to polyvinylidene fluoride (PVDF) membrane (Shanghai Yeasen Biotechnology Co., Ltd., China) by wet-transfer method. Confining liquid with 5% skim milk powder was used to block the membrane with proteins for 1 h. Rabbit anti-rat PI3K and mTOR protein monoclonal antibodies (dilution ratio of 1:800 and 1:1,400 respectively) (Beijing Bioss Biotech Co., Ltd., China) were added, placed at 4°C for incubation overnight. After tris buffered saline with tween (TBST) washing the membrane, HRP-labeled sheep anti-rabbit IgG secondary antibody (dilution ratio of 1:2,000) (Beijing Bioss Biotech Co., Ltd., China) was incubated with the membrane for 1 h. Electrogenerated chemiluminescence (ECL) was used to detect labelled proteins on the membrane. Exposure and photography were performed, followed by film analysis. Image J software was used to measure the gray values of selected protein stripes.

### 2.6 Protective effects of miR-182 on cerebral ischemic oxidative stress model rats

Thirty 10-week-old clean-grade male Wistar rats (Beijing Vital River Laboratory Animal Technology Co., Ltd., China) were purchased with a body weight of 250-300 g, feeding temperature of 22-25 degrees and relative humidity of 45%-55% (license number: SCXK2004-2005). Rats were divided into blank control group, model control group and miR-182 group, with 10 rats in each group. The rats of miR-182 group were injected with miR-182 (15 ng/d) via tail vein injection continuously for 7 days, and the rats of the other two groups were given free diet. On the 8th day, a model of global cerebral ischemia was established by modified Longa method in model control group and miR-182 group. After 90 min of ischemia in rats, reperfusion was performed for 24 h. After anesthesia, blood was collected from the abdominal aorta and rats were killed by cervical dislocation. Hippocampal sections were collected and hippocampal neuron cells were isolated to detect cell viability, apoptosis levels, serum SOD and MDA levels. Hippocampal sections were prepared following paraffin-embedded treatment. Sections were cut to a thickness of 5 μm, and then placed in a 60°C incubator for 2 h. Xylene (Shanghai Yeasen Biotechnology Co., Ltd., China) was used for dewaxing and hydration, then incubated for 10 min in a 3% solution of H_2_O_2_. PBS was used to rinse the samples, and pH 6.0 citrate buffer (Shanghai Yeasen Biotechnology Co., Ltd., China) was added to the tissue. Tissue samples were boiled for 15 min. After cooling, 5% goat serum (Sangon Biotech (Shanghai) Co., Ltd., China) was dropped and then closed for 1 h. After rinsing with PBS, rabbit anti-rat PI3K and mTOR protein primary antibodies (Beijing Bioss Biotech Co., Ltd., China) were added to the tissue, and incubated at room temperature for 2 h. PBS washes were carried out to remove primary antibody from the samples, and sheep anti-rabbit IgG secondary antibody (Beijing Bioss Biotech Co., Ltd., China) was added for incubation at room temperature for 1 h. Diaminobenzidine (DAB) (Sangon Biotech (Shanghai) Co., Ltd., China) was added for straining following the secondary antibody washes. Tissue samples were re-strained, dehydrated and hyalinized, followed by application of a neutral gum seal before being placed under the microscope (Olympus Corporation, Japan) for observation and documentation.

**Ethical approval** The research related to animal use has complied with all the relevant national regulations and institutional policies for the care and use of animals.

### 2.7 Statistical analysis

SPSS19.0 software was used to analyze data. The measurement data were expressed as mean ± standard deviation $\left(\bar{x}\pm \text{sd}\right).$One-way ANOVA was used for comparison of multiple samples. Dunnett’s post hoc t test was used for comparison between groups. P<0.05 indicated statistically significant differences.

## 3 Results

### 3.1 The effect of miR-182 transfection on survival rates and apoptosis of RHiN cells induced by H_2_O_2_

**Figure 1:**
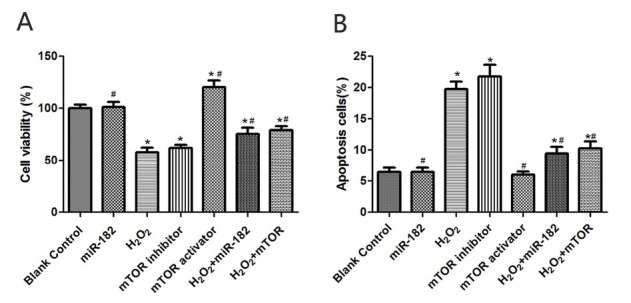
The survival rate and apoptosis of RhiN in each group was determined. A: The survival rates of RHiN in each group. B: The apoptosis rates of RHiN in each group. Compared with balnk control group, ^*^P<0.05; compared with H_2_O_2_ group, ^#^P<0.05. RhiN: rat hippocampal neurons.

The survival rates of RHiN cells in miR-182 group and mTOR activator group were 117.56±6.75% and 116.48±7.28%, significantly higher than blank control group (both P<0.05). The survival rates of RHiN cells in H_2_O_2_ group and mTOR inhibitor group were 57.62±3.94% and 64.39±4.95%, significantly lower than that in blank control group (both P<0.05). The survival rates of RHiN cells in H_2_O_2_+miR-182 group and H_2_O_2_+mTOR group were 73.64±5.37% and 78.13±6.51% respectively, significantly higher than that observed in H_2_O_2_ group (both P<0.05). Flow cytometry analysis indicated that the apoptosis rate of RHiN cells in H_2_O_2_ group was 22.36±3.15%, significantly higher than blank control group (P<0.05). The apoptosis rate of RHiN cells in miR-182 group was 4.27±1.12%, significantly lower than blank control group (P<0.05). The apoptosis rates of RHiN cells in H_2_O_2_+miR-182 group and H_2_O_2_+mTOR group were 8.14±2.18% and 9.32±2.43% respectively, significantly lower than that in H_2_O_2_ group (both P<0.05), as shown in Figure 1.

### 3.2 Effect of miR-182 transfection on SOD and MDA in RHiN cells

**Figure 2:**
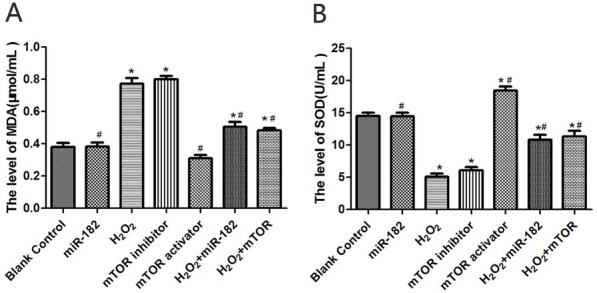
MDA levels and SOD levels in RhiN. A: MDA levels in RHiN of each group. B: SOD levels in RHiN of each group. Compared with balnk control group, ^*^P<0.05; compared with H_2_O_2_ group, ^#^P<0.05. MDA: malondialdehyde; SOD: superoxide dismutase; RhiN: rat hippocampal neurons.

MDA levels in RHiN cells from miR-182 group and mTOR activator group were 0.34±0.03 μmol/mL and 0.31±0.05 μmol/mL, significantly lower than blank control group (both P<0.05). MDA levels in RHiN cells from H_2_O_2_ group and mTOR inhibitor group were 0.75±0.08 μmol/mL and 0.79±0.07 μmol/mL, significantly higher than blank control group (both P<0.05). MDA levels in RHiN cells from H_2_O_2_+miR-182 group and H_2_O_2_+mTOR group were 0.47±0.05 μmol/mL and 0.43±0.04 μmol/mL, significantly lower than H_2_O_2_ group (both P<0.05). SOD levels in RHiN cells from miR-182 group and mTOR activator group were 17.75±0.49 U/mL and 18.14±0.74 U/mL, significantly higher than blank control group (both P<0.05). SOD levels in RHiN cells from H_2_O_2_ group and mTOR inhibitor group were 5.34±0.12 U/mL and 6.04±0.16 U/mL, significantly lower than blank control group (both P<0.05). SOD levels in RHiN cells from H_2_O_2_+miR-182 group and H_2_O_2_+mTOR group were 11.42±0.42 U/mL and 12.37±0.62 U/mL, significantly higher than those in H_2_O_2_ group (both P<0.05), as illustrated in Figure 2.

### 3.3 Effect of miR-182 transfection on mTOR and PI3K mRNA levels in RHiN cells

**Figure 3:**
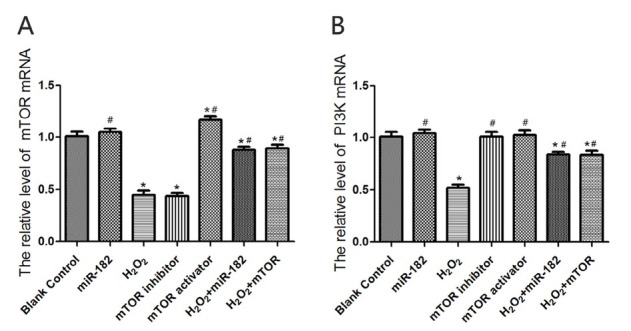
mTOR and PI3K mRNA levels in RHiN. A: The mTOR mRNA levels in hippocampal neurons in each group. B: The PI3K mRNA levels in hippocampal neurons in each group. Compared with balnk control group, ^*^P<0.05; compared with H_2_O_2_ group, ^#^P<0.05. mTOR: mammalian target of rapamycin; PI3K: phosphatidylinositol 3-kinase; RhiN: rat hippocampal neurons.

RT-PCR experiments showed that the mTOR mRNA level in RHiN cells from mTOR activator group was 1.17±0.19, higher than blank control group (P<0.05). The mTOR mRNA levels in RHiN cells from H_2_O_2_ group and mTOR inhibitor group were 0.64±0.08 and 0.43±0.06, lower than blank control group (both P<0.05). The mTOR mRNA levels in RHiN cells from H_2_O_2_+miR-182 group and H_2_O_2_+mTOR group were 0.82±0.13 and 0.85+0.14, significantly higher than H_2_O_2_ group (both P<0.05). The PI3K mRNA level in miR-182 group was 1.13±0.03, significantly higher than H_2_O_2_ group (P<0.05). The PI3K mRNA level in H_2_O_2_ group was 0.48±0.04, significantly lower than blank control group (P<0.05). The PI3K mRNA levels in H_2_O_2_+miR-182 group and H_2_O_2_+mTOR group were 0.87±0.06 and 0.89±0.09, which were significantly higher than those in H_2_O_2_ group (both P<0.05). Comparisons of the mRNA expression levels are shown in Figure 3.

### 3.4 The effect of miR-182 on mTOR and PI3K protein levels in RHiN cells

**Figure 4:**
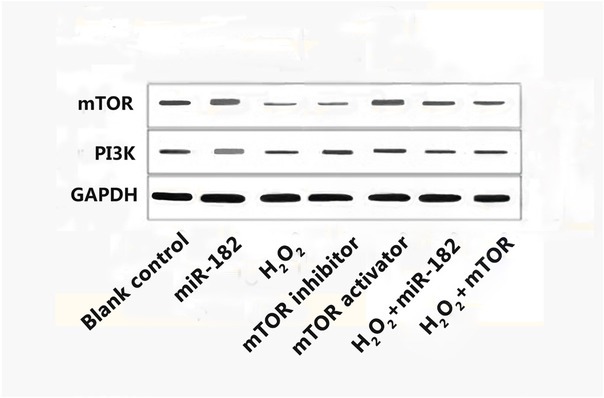
Electrophoretogram of mTOR and PI3K in RHiN. mTOR: mammalian target of rapamycin; PI3K: phosphatidylinositol 3-kinase; GAPDH: Glyceraldehyde-3-phosphate dehydrogenase; RhiN: rat hippocampal neurons.

**Figure 5:**
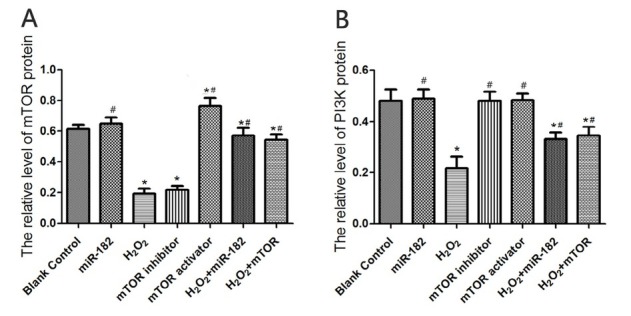
mTOR and PI3K protein levels in RHiN cells. A: Values of expression levels of mTOR protein in each group. B: Values of expression levels of PI3K protein in each group. Compared with blank control group, ^*^P<0.05; compared with H_2_O_2_ group, ^#^P<0.05. mTOR: mammalian target of rapamycin; PI3K: phosphatidylinositol 3-kinase; RhiN: rat hippocampal neurons.

Western Blot evaluation of protein expressions determined that the expression level of mTOR protein in mTOR activator group was 0.71±0.18, higher than blank control group (P<0.05). The expression levels of mTOR protein in H_2_O_2_ group and mTOR inhibitor group were 0.18±0.03 and 0.23±0.04, lower than blank control group (both P<0.05). The expression levels of mTOR protein in H_2_O_2_+miR-182 group and H_2_O_2_+mTOR group were 0.45±0.06 and 0.42±0.04, significantly higher than H_2_O_2_ group (both P<0.05). The expression level of PI3K protein in miR-182 group was 0.57±0.08, significantly higher than H_2_O_2_ group (P<0.05). The expression level of PI3K protein in H_2_O_2_ group was 0.23±0.06, significantly lower than blank control group (P<0.05). The expression levels of PI3K protein in H_2_O_2_+miR-182 group and H_2_O_2_+mTOR group were 0.36±0.04 and 0.38±0.05, significantly higher than H_2_O_2_ group (both P<0.05). Comparisons of the protein expression levels are shown in Figure 4 and Figure 5.

### 3.5 Protective effects of miR-182 on cerebral ischemic oxidative stress model rats

**Figure 6:**
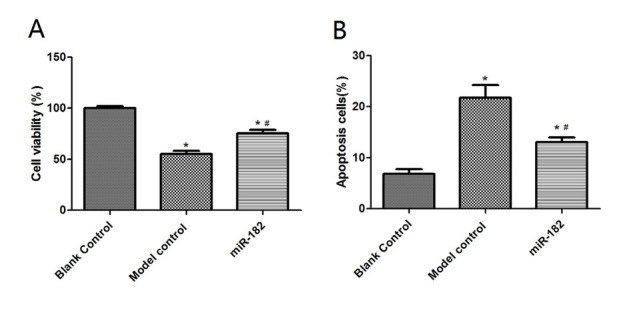
The survival rate and apoptosis of rat hippocampal neuronal cells in each group. A: The survival rate of rat hippocampal neuronal cells. B: The apoptosis of rat hippocampal neuronal cells. Compared with blank control group, ^*^P<0.05; compared with H_2_O_2_ group, ^#^P<0.05.

**Figure 7:**
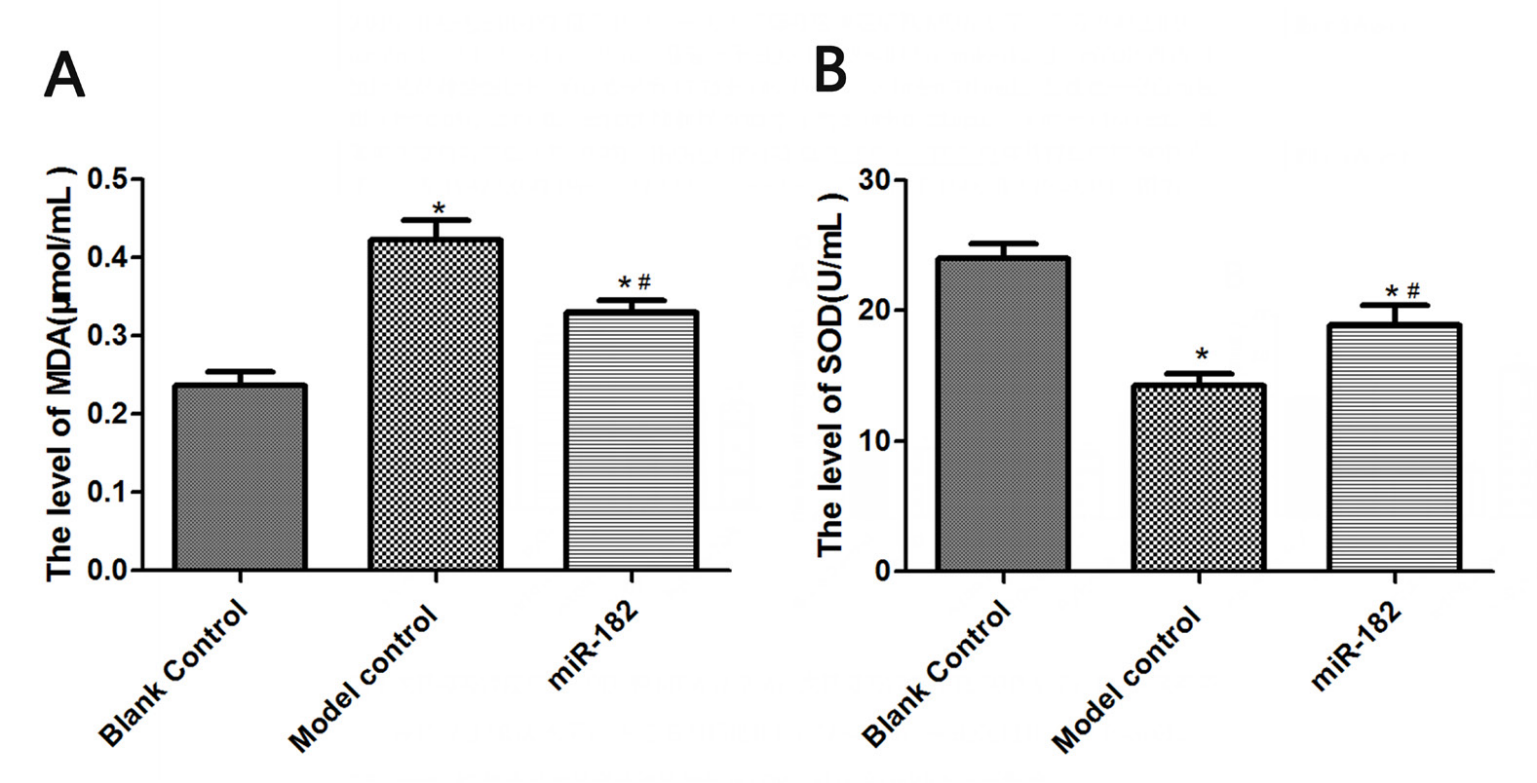
MDA levels and SOD levels in serum of rats. A: MDA levels in rat hippocampal neuronal cells. B: SOD levels in rat hippocampal neuronal cells. Compared with balnk control group, ^*^P<0.05; compared with model control group, ^#^P<0.05. MDA: malondialdehyde; SOD: superoxide dismutase.

**Figure 8:**
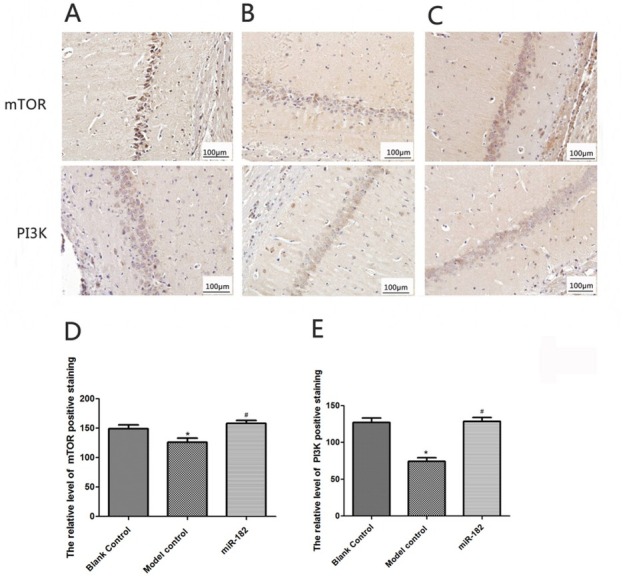
Immunohistochemistry staining of rat hippocampus tissue (200X). A: mTOR and PI3K protein staining in rat hippocampus tissue in blank control group. B: mTOR and PI3K protein staining in rat hippocampus tissue in model control group. C: mTOR and PI3K protein staining in rat hippocampus tissue in miR-182 group. D: The positive staining intensity of mTOR protein in rat hippocampus tissue of each group. E: The positive staining intensity of PI3K in rat hippocampus tissue of each group. Compared with blank control group, ^*^P<0.05; compared with model control group, ^#^P<0.05. mTOR: mammalian target of rapamycin; PI3K: phosphatidylinositol 3-kinase.

The survival rate and SOD level of rat hippocampal neuronal cells in miR-182 group were 62.15±4.28% and 11.36±2.41 U/mL respectively, significantly higher than model control group (both P<0.05). The apoptosis rate and MDA level of rat hippocampal neuronal cells in miR-182 group were 13.12±2.16% and 0.41±0.05 μmol/mL, significantly lower than model control group (both P<0.05). Immunohistochemical staining showed that the intensities of mTOR and PI3K in model group were 124.57±18.43 and 87.26±18.43 respectively, significantly lower than blank control group (P<0.05). Positive staining intensities of mTOR and PI3K were 141.25±16.52 and 124.18±19.49 respectively in miR-182 group, which were significantly higher than that in model blank group (both P<0.05), as shown in Figure 6, Figure 7 and Figure 8.

## 4 Discussion

The hippocampus is central to information storage and memory functions in the brain. Oxidative stress injury to RHiN cells can lead to neurological deficits in the brain. Furthermore, oxidative injuries may cause various neurodegenerative diseases such as Alzheimerʼs disease and Parkinsonʼs disease [8]. Atalay et al. have found that the memory and neural activity of rats decreases significantly after oxidative stress in RHiN cells [9]. The apoptosis rate of neurons increased after the increase in oxidative stress in the hippocampus, and the rats appeared to suffer from cognitive impairment [10]. When oxygen free radicals (such as ROS and NOS) cannot be effectively removed from cells, active constituents such as DNA, protein and other macromolecules are attacked. Further, the potential of the mitochondrial membrane is changed, and neuronal apoptosis is induced as a result [11, 12]. Therefore, effective enhancement of the antioxidant capacity of neurons is of great value in reducing oxidative stress injury.

In this study, cell viability of RHiN cells in the H_2_O_2_+miR-182 group was increased after miR-182 transfection, as demonstrated by effective reduction of cellular apoptosis. Apoptosis results indicate that miR-182 is involved in regulating the oxidative stress response of rat neurons, which effectively reduces the damage incurred from oxidative stress. SOD is an enzyme that can effectively remove free radicals *in vivo*, thereby influencing cellular antioxidant levels. When the levels of SOD are reduced, there are excess free radicals without an effective removal system. Oxidative damage of brain neurons from this excess of radicals has been reported [13, 14]. MDA is the end-product of cells attacked by free radicals. It is cytotoxic and can exacerbate damage to cellular functions [15, 16, 17]. Zhang et al. reported that SOD levels of retinal ganglion cells are significantly reduced after oxidative stress damage, and the reduction is associated with excitatory transmission dysfunction of the neurons [18]. In this experiment, SOD levels in treated RHiN cells were decreased after H_2_O_2_ treatment and MDA levels were increased, suggesting higher levels of oxidative stress compared to controls. After transfection of miR-182 in rat neurons, the levels of SOD increased and MDA levels decreased. This affect indicates that miR-182 transfection alleviates oxidative stress in neuronal cells, which is consistent with previous studies [19].

The PI3K-AKT-mTOR signaling pathway plays an important role in regulating cell growth, apoptosis, autophagy and cellular antioxidant capacity. PI3K is a key protein in the signaling pathway, as it can be phosphorylated to form phospholipid creatine phosphate, thereby prompting the phosphorylation of AKT kinase [20]. mTOR is a serine/threonine protein kinase that regulates the activity of ribosomal S6 protein kinase (S6K) and eukaryotic promoter 4E binding protein 1 (4E-BP1), which further act to regulate downstream gene transcription and protein synthesis [21, 22, 23]. Jiao et al. have found that increased levels of mTOR and PI3K proteins in cardiac myocytes can increase antioxidant levels in the cells. The higher levels are associated with alleviating free radical damage, thus enhancing the cells’ viability under adverse conditions [1]. Huang W et al. have also found that the myocytes are sensitive to oxidative stress and prone to apoptosis after treatment with an mTOR-specific antagonist [24]. The results of this study demonstrate the intensified oxidative stress reaction of RHiN cells after treatment with H_2_O_2_. The expression levels of mTOR and PI3K proteins were decreased as a result of the peroxide challenge. Transfection of miR-182 increased the expression of mTOR and PI3K proteins in rat neurons and activated the PI3K-AKT-mTOR signaling pathway, with its concomitant positive effects on relieving neuronal oxidative stress. Our results suggest that miR-182 increases the antioxidant capacity of neuronal cells. At the same time, our study showed that miR-182 could alleviate the damage of oxidative stress on rat hippocampal neuronal cells in cerebral ischemic oxidative stress model rats. The mechanism of action of miR-182’s influence on the expression of mTOR and PI3K in rat neurons is beyond the scope of this study, however our initial indications warrant further investigations of related mechanisms.

In conclusion, miR-182 can effectively alleviate oxidative damage to rat hippocampal neurons induced by H_2_O_2_. The mechanism of miR-182’s efficacy involves activation of the PI3K-AKT-mTOR signaling pathway as a means for alleviating the damage from free radicals within the neuronal cells.
